# Machine Learning for Electrocardiographic Features to Identify Left Atrial Enlargement in Young Adults: CHIEF Heart Study

**DOI:** 10.3389/fcvm.2022.840585

**Published:** 2022-03-01

**Authors:** Chu-Yu Hsu, Pang-Yen Liu, Shu-Hsin Liu, Younghoon Kwon, Carl J. Lavie, Gen-Min Lin

**Affiliations:** ^1^Department of Medicine, Hualien Armed Forces General Hospital, Hualien City, Taiwan; ^2^Department of Internal Medicine, Tri-Service General Hospital and National Defense Medical Center, Taipei City, Taiwan; ^3^Department of Medicine, Taoyuan Armed Forces General Hospital, Taoyuan City, Taiwan; ^4^Department of Nuclear Medicine, Hualien Tzu Chi Hospital, Hualien City, Taiwan; ^5^Department of Internal Medicine, University of Washington, Seattle, WA, United States; ^6^John Ochsner Heart and Vascular Institute, Ochsner Clinical School, The University of Queensland School of Medicine, New Orleans, LA, United States

**Keywords:** echocardiography, electrocardiography, machine learning, left atrial enlargement, young adults

## Abstract

**Background:**

Left atrial enlargement (LAE) is associated with cardiovascular events. Machine learning for ECG parameters to predict LAE has been performed in middle- and old-aged individuals but has not been performed in young adults.

**Methods:**

In a sample of 2,206 male adults aged 17–43 years, three machine learning classifiers, multilayer perceptron (MLP), logistic regression (LR), and support vector machine (SVM) for 26 ECG features with or without 6 biological features (age, body height, body weight, waist circumference, and systolic and diastolic blood pressure) were compared with the P wave duration of lead II, the traditional ECG criterion for LAE. The definition of LAE is based on an echocardiographic left atrial dimension > 4 cm in the parasternal long axis window.

**Results:**

The greatest area under the receiver operating characteristic curve is present in machine learning of the SVM for ECG only (77.87%) and of the MLP for all biological and ECG features (81.01%), both of which are superior to the P wave duration (62.19%). If the sensitivity is fixed to 70–75%, the specificity of the SVM for ECG only is up to 72.4%, and that of the MLP for all biological and ECG features is increased to 81.1%, both of which are higher than 48.8% by the P wave duration.

**Conclusions:**

This study suggests that machine learning is a reliable method for ECG and biological features to predict LAE in young adults. The proposed MLP, LR, and SVM methods provide early detection of LAE in young adults and are helpful to take preventive action on cardiovascular diseases.

## Introduction

Machine learning, an artificial intelligence (AI)-based computational statistic, has been broadly applied to clinical practice in medicine to assess disease risk and diagnosis (1–12). For instance, Lin et al. (12) used the support vector machine (SVM) classifier for some ECG features training successfully to identify echocardiographic left ventricular hypertrophy, and the performance of SVM was superior to the conventional ECG voltage criteria. In the modern age, the impact of machine learning is tremendously growing in medicine and has become a cost-effective and practical tool for physicians.

Left atrial enlargement (LAE) is related to high blood volume status (i.e., mitral regurgitation and elite endurance athletes) (13, 14) and elevated left ventricular (LV) diastolic pressure (i.e., obesity, hypertension, and great LV mass) (15–17). LAE is a precursor of left atrial dysfunction and has been associated with incident atrial fibrillation, ischemic stroke, and cardiovascular events in middle- and old-aged individuals (18–21). The prevalence of LAE is increased with aging, (18) and, in young adults, LAE is usually observed in those undergoing rigorous physical training, particularly accumulated lifetime training >3,600 h (22–24). A prior coronary artery disease risk development in young adults (CARDIA) study also revealed that the presence of LAE at a young age is a risk factor in incident cardiovascular events occurring in midlife (25). Therefore, early detection of LAE is vital to prevent the development of cardiovascular diseases and related sequelae.

The P wave duration in lead II ≥ 120 milliseconds is currently the most commonly used ECG criterion for the general population to screen for the presence of echocardiographic LAE, which is mainly defined as a diastolic left atrium dimension >4 cm in the parasternal long axis window (26). The P wave duration was also a predictor of atrial fibrillation, cardiovascular death, and early vascular aging (27, 28). Over the past 5 years, there were only some hospital-based studies utilizing machine learning for ECG features to detect the presence of LAE, in which the area under the curve (AUC) of the receiver operating characteristic curve (ROC) varied much from 0.62 to 0.98 (29–31). However, there were no previous reports performed in the general population. The aim of the study was to investigate the performance of machine learning for ECG features to identify LAE in a military cohort of young male adults.

## Methods

### Study Population

A population of 2,268 military males aged 17–43 years were obtained from the cardiorespiratory health in eastern armed forces study (CHIEF Heart Study) for the machine learning experiment (32–35). All the participants received the annual health examination for their demographic, anthropometric, and hemodynamic measurements in the Hualien Armed Forces General Hospital of Taiwan from 2016 to 2021. Anthropometric parameters, including body height, weight, and waist circumference, of each participant were measured in the standing position. The hemodynamic parameter for blood pressure of each participant was measured one time over the right upper arm in a sitting position after at least 15 min of rest by an automatic oscillometric monitor (PARAMA TECH FT-201, Fukuoka, Japan). In addition, all the participants received 12-lead ECG and echocardiography to assess their cardiac structure and function during the same period. Sixty-two participants were excluded for a lack of relevant data (*n* = 36) or were unwilling to sign informed consent (*n* = 26), leaving a sample of 2,206 males for analysis.

### ECG and Echocardiographic Measurements

A 12-lead ECG was performed for each participant (Schiller AG CARDIOVIT MS-2015, Baar, Switzerland). If the quality of the ECG report was not interpretable (i.e., baseline wandering), a new ECG would be repeated by the technician. The analysis for the ECG parameters, such as the heart rate and P-QRS-T wave duration or interval, was performed by the software in the ECG machine and interpreted by a board-certified cardiologist.

Transthoracic echocardiography using a 1–5 MHz transducer (iE33; Philips Medical Systems, Andover, MA, USA) was performed following the ECG procedure at the Hualien-Armed Forces General Hospital. Measurements of left atrial dimensions were based on the recommendations of the American Society of Echocardiography (36). LAE was defined as the left atrial diameter in the image of 2-D or M-mode > 4 cm, which was calculated from the posterior aortic wall to the posterior left atrial wall for men in the parasternal long-axis view at the end-ventricular systole. The prevalence of LAE in the young males was 4.85% (107/2206). The profiles of those with and without LAE are shown in Table 1 and compared by ANOVA, where a *p* < 0.05 was considered significant. The study design and protocol were approved by the Institutional Review Board of Mennonite Christian Hospital (No. 16-05-008) in Hualien City, Taiwan.

**Table 1:** Comparisons in electrocardiographic and biological features between participants with and without left atrial enlargement.

**Clinical features**	**Non-LAE**	**LAE**	**Total**	***P-Value***
	N = 2099	N = 107	N = 2206	
Age (years)	27.88 ± 6.14	30.16 ± 6.28	27.99 ± 6.17	0.0002
Height (cm)	172.06 ± 5.73	173.98 ± 6.25	172.16 ± 5.77	0.0008
Weight (kg)	73.44 ± 11.87	84.92 ± 15.19	74.00 ± 12.30	<0.0001
Waist size (cm)	83.38 ± 9.57	92.16 ± 12.17	83.80 ± 9.89	<0.0001
Systolic BP (mmHg)	119.30 ± 13.14	126.40 ± 16.92	119.65 ± 13.43	<0.0001
Diastolic BP (mmHg)	70.81 ± 10.24	76.00 ± 14.19	71.06 ± 10.52	0.0003
Heart rate (bpm)	66.51 ± 10.87	66.35 ± 10.58	66.50 ± 10.85	0.8785
P duration-II (ms)	106.68 ± 14.51	106.31 ± 18.09	106.67 ± 14.70	0.8344
PR interval-II (ms)	157.70 ± 20.11	159.72 ± 20.53	157.80 ± 20.13	0.3114
QRS duration-II (ms)	97.58 ± 10.61	99.76 ± 14.18	97.69 ± 10.81	0.1206
QT interval-II (ms)	372.31 ± 27.68	379.59 ± 28.59	372.67 ± 27.77	0.0082
P axis-II (degree)	44.57 ± 24.43	37.12 ± 27.65	44.21 ± 24.64	0.0023
QRS-II (degree)	62.19 ± 31.26	50.62 ± 42.88	61.63 ± 32.00	0.0068
T axis-II (degree)	34.10 ± 19.77	28.49 ± 31.57	33.82 ± 20.52	0.0715
R-I (mm)	5.98 ± 2.97	7.90 ± 3.85	6.07 ± 3.04	<0.0001
R-II (mm)	12.51 ± 4.92	10.92 ± 4.92	12.43 ± 4.93	0.0011
R-III (mm)	7.88 ± 5.67	6.27 ± 5.25	7.80 ± 5.66	0.0042
R-aVR (mm)	1.31 ± 1.78	1.63 ± 2.38	1.32 ± 1.81	0.1671
R-aVL (mm)	2.94 ± 2.41	4.91 ± 3.63	3.04 ± 2.52	<0.0001
R-aVF (mm)	10.00 ± 5.22	7.92 ± 5.36	9.90± 5.24	0.0001
R-V1 (mm)	3.40 ± 2.09	3.48 ± 2.78	3.40 ± 2.13	0.752
S-V1 (mm)	9.94 ± 5.08	8.63 ± 4.41	9.88± 5.05	0.0089
R-V2 (mm)	8.54 ± 4.05	9.32 ± 4.41	8.58 ± 4.07	0.0537
S-V2 (mm)	15.46 ± 6.63	14.21 ± 6.53	15.40 ± 6.63	0.0579
R-V3 (mm)	12.84 ± 5.64	13.76 ± 6.32	12.89 ± 5.68	0.1042
S-V3 (mm)	8.51 ± 5.17	8.70 ± 5.07	8.51 ± 5.16	0.6968
R-V4 (mm)	19.11 ± 6.66	17.93 ± 6.48	19.06 ± 6.66	0.072
S-V4 (mm)	5.44 ± 4.01	6.13 ± 4.14	5.47 ± 4.02	0.0842
R-V5 (mm)	19.81 ± 5.83	19.13 ± 5.37	19.78 ± 5.81	0.2386
S-V5 (mm)	3.46 ± 2.90	3.98 ± 3.33	3.49 ± 2.92	0.1166
R-V6 (mm)	16.78 ± 4.96	16.54 ± 4.65	16.77 ± 4.94	0.6256
S-V6 (mm)	2.11 ± 1.98	2.48 ± 2.48	2.12 ± 2.01	0.1293

*BP, blood pressure; LAE, left atrial enlargement*.

### Machine Learning Procedures

Three machine learning classifiers, including the multilayer perceptron (MLP) (37), logistic regression (LR) (38), and support vector machine (SVM) with a linear kernel (39), were used for 26 ECG features (heart rate; P wave duration in lead II; intervals of PR, QRS, and QT in lead II; axes of P, QRS, and T waves in lead II; voltages of the R wave in limb leads I, II, III, aVR, aVL, and aVF; voltages of both the R and S waves in precordial leads V1–V6) and with or without six biological features (age, body height, body weight, waist circumference, and systolic and diastolic blood pressure) training to identify the presence of LAE from military young males in Taiwan. The normalization of Min–Max scaling was used for the input data to execute a linear transformation (40). The original data of all 32 ECG and biological features were adjusted to a normalized value between 0 and 1. The MLP model includes an input layer, hidden layers, and an output layer (37). In hidden layers, the rectified linear unit (ReLU) activation function is utilized for each node, and the logistic regression function is used to determine the output layer. LR is a linear model that transforms its output using the logistic sigmoid function to return a probability value (38). The loss function includes the loss term and the regularization term. The loss term for learning the weight vector is negative log-likelihood, and the regularization term is used to avoid overfitting. In SVM (39), the maximum margin is constructed to maximize the distance from the hyperplane to the nearest subset of the training data points (support vectors) of the LAE or non-LAE class. The soft-margin SVM with regularization technique weighted by hyperparameter is adopted to allow the wide decision margin (39). The optimized hyperparameters for the three machine learning classifiers are chosen by grid search based on the average AUC of the ROC curves of the cross validation.

#### Data Augmentation and Cross Validation

**Figure 1:**
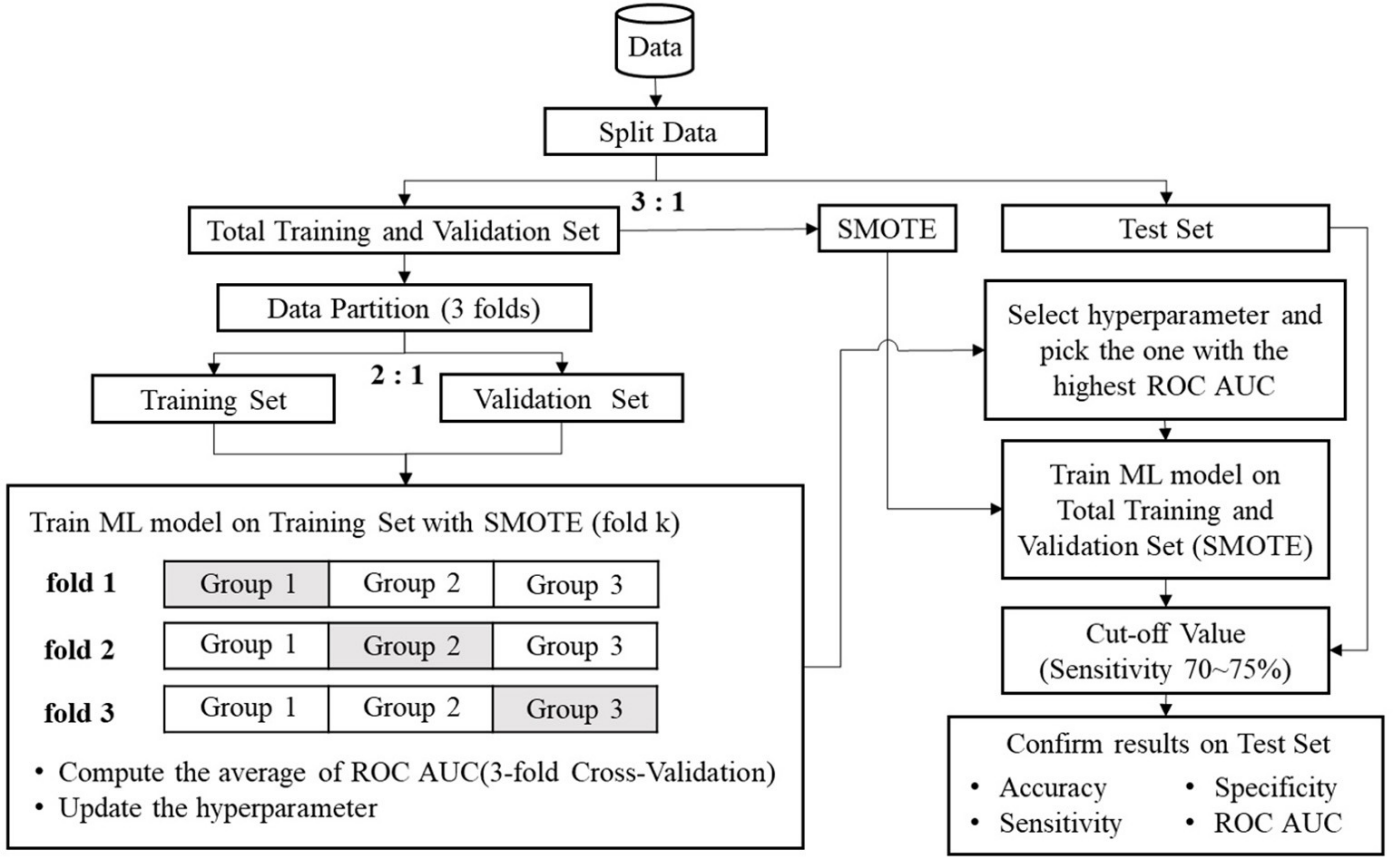
The flow chart of the proposed methods.

The data of the 2,206 participants were randomly grouped by a 3:1 ratio into a training/validation set (*n* = 1,654) and a test set (*n* = 552). Three subgroups of equal size were divided from the training/validation set. Two subgroups of the training/validation set were used for training, and the remaining subgroup was used for validation. The data numbers illustrated by the three folds are shown in Table 2. Because there was an imbalance in sample size between LAE and non-LAE cases, the synthetic minority oversampling technique (SMOTE) (41) was applied to artificially augment the LAE cases. Using the SMOTE to create sufficient new minority class cases, a near neighbor of the minority class of the index cases was randomly chosen for interpolation. The decision space for the LAE cases was magnified, and the SMOTE method could balance the number of each category. After data augmentation, the three subgroups were replaced to repeat the process: two for training and one for validation. An average of the three AUCs of the ROC curves from the 3-fold cross validations was treated as a single performance. The raw data that were not preprocessed by SMOTE for machine learning were used to confirm the validity of SMOTE. This study utilized scikit learn v0.20.2 software and Python programming language for the proposed methods. The flow chart for data preprocessing and machine learning is shown in Figure 1.

**Table 2:** Data numbers in the dataset.

**Fold**	**Data**	**Non-LAE**	**LAE**	**Total**
	Training set	1047	55	1102
1st	Pre-processed by SMOTE	1047	1047	2094
	Validation set	519	33	552
	Training set	1037	66	1103
2nd	Pre-processed by SMOTE	1037	1037	2074
	Validation set	529	22	551
	Training set	1048	55	1103
3rd	Pre-processed by SMOTE	1048	1048	2096
	Validation set	518	33	551
	Total training/Validation set	1566	88	1654
	Pre-processed by SMOTE	1566	1566	3132
	Testing set	533	19	552

*LAE, left atrial enlargement; SMOTE, synthetic minority over-sampling technique*.

## Results

### Comparisons of the Performance in Machine Learning Classifiers

**Figure 2:**
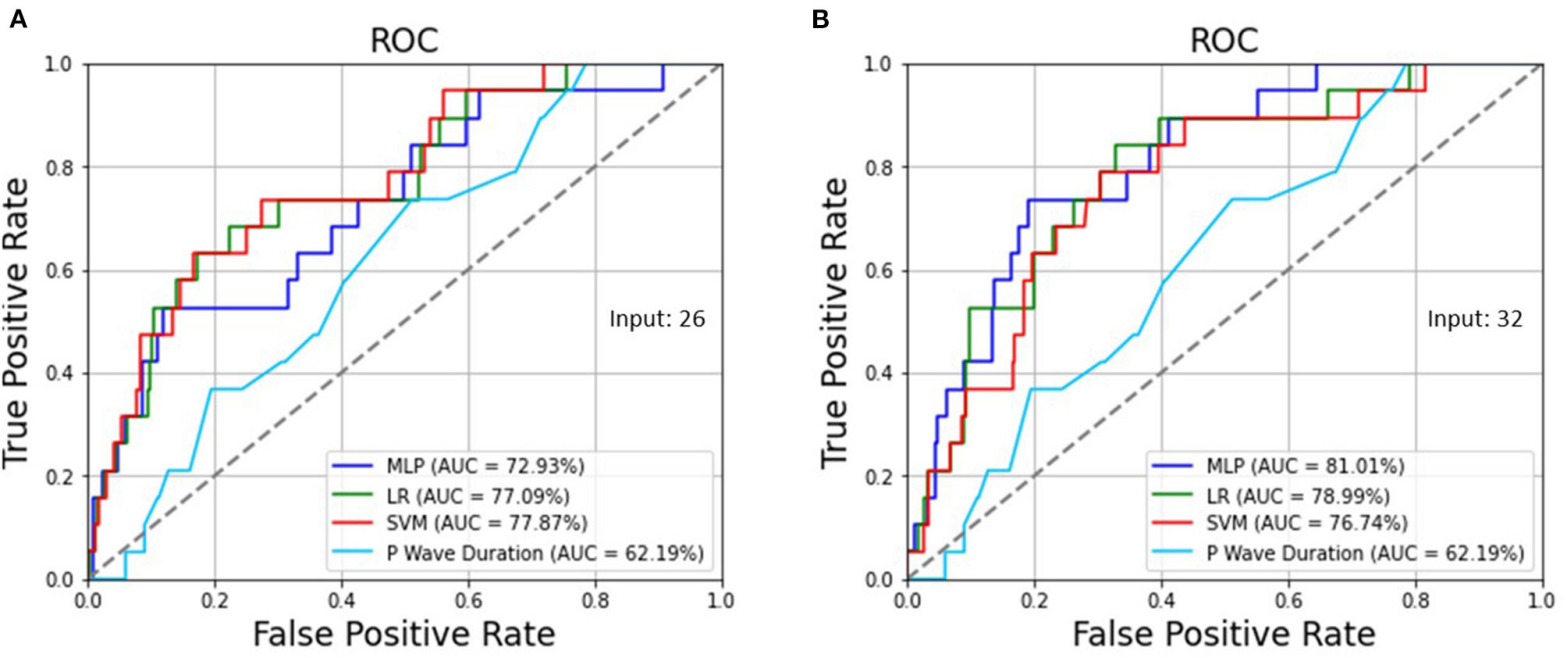
**(A)** The AUCs of the ROC curves were 72.93, 77.09, and 77.87% using the MLP, LR, and SVM, respectively, for 26 ECG features as well as **(B)** 81.01, 78.99, and 76.74% utilizing the MLP, LR, and SVM, respectively, for 32 ECG and biological features, which are >62.19% for the P wave duration in lead II.

Table 3 demonstrates the optimized hyperparameters for the machine learning methods in the testing set. Regarding the MLP, LR, and SVM for 26 ECG features preprocessed by SMOTE, when the sensitivity was 73.68% for all, the specificity was 57.41, 69.79, and 72.42%, respectively, and the accuracy was 57.97, 69.93, and 72.46%, respectively, as shown in Table 4, which were superior to those without SMOTE in Supplementary Table 1. Regarding the MLP, LR, and SVM for all 32 ECG and biological features, when the sensitivity was 73.68% for all, the specificity was 81.05, 73.73, and 71.67%, and the accuracy was 80.80, 73.73, and 71.74%, respectively. While the traditional ECG criterion of the P wave duration in lead II was ≥ 106 ms for LAE, the sensitivity, specificity, and accuracy were 73.68, 48.78, and 49.64%, respectively. The AUCs of the ROC curves shown in Figure 2 were 72.93, 77.09, and 77.87% using the MLP, LR, and SVM, respectively, for 26 ECG features and 81.01, 78.99, and 76.74% utilizing the MLP, LR, and SVM, respectively, for 32 ECG and biological features, which were much >62.19% for the P wave duration in lead II.

**Table 3:** Hyperparameter optimization.

	**Hyperparameter**	**Beginning value**	**Ending value**	**Interval**	**Optimal value (32 features)**	**Optimal value (26 features)**
	Regularization	1.0	15.0	1.0	6	3
MLP	Number of hidden layers	-	-	-	3	4
	Number of neurons	-	-	-	20, 10, 5	20, 10, 5
	Number of iterations	-	-	-	10,000	10,000
LR	Regularization	0.001	1.000	0.001	0.694	0.524
SVM	Regularization	0.01	1.00	0.01	0.9	0.29

*LR, logistic regression; MLP, multilayer perceptron; SVM, support vector machine*.

**Table 4:** Performance comparisons of proposed methods and previous works.

	**Sensitivity**	**Specificity**	**Accuracy**	**AUC of ROC**	**TN**	**FN**	**TP**	**FP**
MLP (Input 32)	73.68%	**81.05%**	**80.80%**	**81.01%**	432	5	14	101
LR (Input 32)	73.68%	73.73%	73.73%	78.99%	393	5	14	140
SVM (Input 32)	73.68%	71.67%	71.74%	76.74%	382	5	14	151
MLP (Input 26)	73.68%	57.41%	57.97%	72.93%	306	5	14	227
LR (Input 26)	73.68%	69.79%	69.93%	77.09%	372	5	14	161
SVM (Input 26)	73.68%	**72.42%**	**72.46%**	**77.87%**	386	5	14	147
P wave duration	21.05%	87.24%	84.96%	62.19%	465	15	4	68
	73.68%	**48.78%**	**49.64%**		260	5	14	273

*AUC, area under curve; LR, logistic regression; FN, false negative; FP, false positive; MLP, multilayer perceptron; ROC, receiver operating characteristic; SVM, support vector machine; TN, true negative; TP, true positive*.

### ECG Features Importance in the SVM Classifier

**Figure 3:**
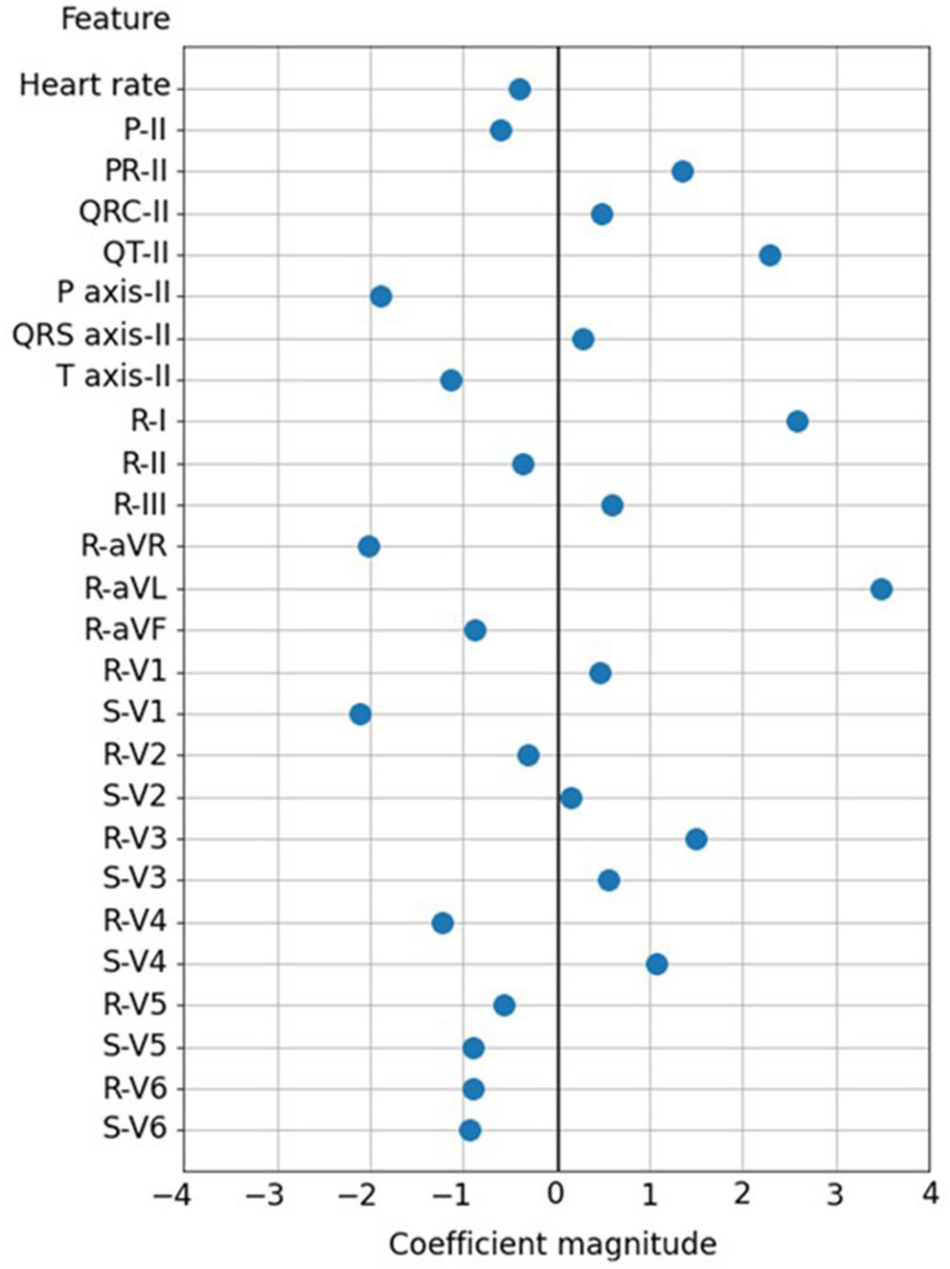
The 26 ECG feature importance in the SVM classifier.

Figure 3 shows the 26 ECG feature importance of the SVM classifier. A greater R wave voltage of leads aVL and I and QT interval of lead II were the most important factors of echocardiographic LAE, with a coefficient magnitude >2 in the SVM model. The other potent predictors of LAE with greater coefficient magnitude included greater S wave voltage of lead V1, P wave axis of lead II, and R wave voltage of lead aVR.

## Discussion

The study was the first report to show a better performance of machine learning to predict echocardiographic LAE compared to the traditional ECG criterion of P wave duration in young male adults who had a healthy status and without multiple comorbidities. Prior studies (29–31) have revealed that machine learning for ECG features could detect most of the LAE cases from hospitalized patients, probably due to those patients with LAE who were likely to have other cardiac comorbidities, such as heart failure, that were easily reflected by ECG features; thus, the results might not be appropriate for healthy individuals.

Some studies have shown that, in young adults, particularly physically fit people, an enlarged cardiac chamber is likely, and the typical ECG features for LAE might not be the same as those in middle-aged individuals and elderly individuals who had several cardiovascular comorbidities, i.e., hypertension. This study revealed that the P wave axis rather than the P wave duration was a strong indicator for LAE. In addition, a greater R wave in leads aVL and I and an S wave in lead V1 representing an enhanced left lateral electrical force in the heart (42) and a greater QT interval representing a longer diastolic phase of electrical repolarization and left ventricular relaxation were vital predictors of LAE. These findings emphasize the necessity of performing machine learning, specifically for physically young adults to identify LAE. The SVM was the best machine learning classifier for ECG features only to detect LAE in young males, achieving an AUC of 78% of the ROC. In contrast, the MLP was the best machine learning classifier, which could improve the performance from 73 to 81% after biological features were added to the MLP model. It was obvious that the addition of biological features did not improve the predictive performance of the SVM and LR classifiers.

### Study Strengths and Limitations

The main strengths of this study included the following: First, military males were physically active, and the training program was conducted in Eastern Taiwan. In addition, since the living environment is a closed system, the participants have a similar daily schedule, and the unmeasured bias could be minimized. Third, this was the first study using machine learning for ECG and biological features to predict LAE early in young adults. In contrast, the data were only obtained from the males, and the results might not be the same for the females. Second, other feature learning methods, such as convolutional neural networks for ECG training to predict LAE, were not performed, which may be a focus of future works. Third, since LAE is highly associated with atrial fibrillation, follow-up studies allow a conclusion related to atrial fibrillation. Finally, oxidative stress was also related to the occurrence of atrial fibrillation (43), and this was not considered in this study.

## Conclusion

This study suggests that it is reliable to use machine learning for ECG features and biological features to predict LAE in young adults. The proposed MLP, LR, and SVM methods could provide early detection of LAE in young adults in clinical settings and may be useful in screening for high-risk groups of young adults for cardiovascular diseases, i.e., atrial fibrillation, which has an important relationship with LAE.

## Data Availability Statement

The original contributions presented in the study are included in the article/Supplementary Material, further inquiries can be directed to the corresponding author/s.

## Ethics Statement

The studies involving human participants were reviewed and approved by Institutional Review Broad of Mennonite Christian Hospital (No. 16-05-008). The patients/participants provided their written informed consent to participate in this study.

## Author Contributions

C-YH wrote the paper. P-YL collected the data. S-HL, YK, and CL raised critical comments for the paper. G-ML analyzed data and edited the manuscript and was the principal investigator for the CHIEF study. All authors contributed to the article and approved the submitted version.

## Funding

This study was supported by the Medical Affairs Bureau Ministry of National Defense and Hualien Armed Forces General Hospital, Taiwan, under the grants MND-MAB-110-148, MND-MAB-D-11115, HAFGH-D-110008, and HAFGH-D-111003.

## Conflict of Interest

The authors declare that the research was conducted in the absence of any commercial or financial relationships that could be construed as a potential conflict of interest.

## Publisher's Note

All claims expressed in this article are solely those of the authors and do not necessarily represent those of their affiliated organizations, or those of the publisher, the editors and the reviewers. Any product that may be evaluated in this article, or claim that may be made by its manufacturer, is not guaranteed or endorsed by the publisher.
