# Supplementary material for: Machine Learning for Electrocardiographic Features to Identify Left Atrial Enlargement in Young Adults: CHIEF Heart Study
Source: Front Cardiovasc Med. 2022 Mar 1;9:840585. doi: 10.3389/fcvm.2022.840585 (PMC8921457; doi:10.3389/fcvm.2022.840585)
Supplement: Supplementary file 1 [file Table_1.docx]

Supplemental Table 1. Performance of Proposed Machine Learning Methods Using Raw Data Without SMOTE Pre-Processed

|  | Sensitivity | | Specificity | Accuracy | AUC of ROC | TN | FN | TP | FP |
| --- | --- | --- | --- | --- | --- | --- | --- | --- | --- |
| MLP (Input 26) | | 73.68% | 41.65% | 42.75% | 68.63% | 222 | 5 | 14 | 311 |
| LR (Input 26) | | 73.68% | 65.29% | 65.58% | 75.20% | 348 | 5 | 14 | 185 |
| SVM (Input 26) | | 73.68% | **56.85%** | **57.43%** | **72.24%** | 303 | 5 | 14 | 230 |

Abbreviations: LR, logistic regression; FN, false negative; FP, false positive; MLP, multilayer perceptron; SVM, support vector machine; TN, true negative; TP, true positive
